# A bottom-up perspective on ecosystem change in Mesozoic oceans

**DOI:** 10.1098/rspb.2016.1755

**Published:** 2016-10-26

**Authors:** Andrew H. Knoll, Michael J. Follows

**Affiliations:** 1 Department of Organismic and Evolutionary Biology, Harvard University, Cambridge, MA 02138, USA; 2 Department of Earth, Atmospheric and Planetary Sciences, Massachusetts Institute of Technology, Cambridge, MA 02139, USA

**Keywords:** phytoplankton, ecosystem model, predation

## Abstract

Mesozoic and Early Cenozoic marine animals across multiple phyla record secular trends in morphology, environmental distribution, and inferred behaviour that are parsimoniously explained in terms of increased selection pressure from durophagous predators. Another systemic change in Mesozoic marine ecosystems, less widely appreciated than the first, may help to explain the observed animal record. Fossils, biomarker molecules, and molecular clocks indicate a major shift in phytoplankton composition, as mixotrophic dinoflagellates, coccolithophorids and, later, diatoms radiated across shelves. Models originally developed to probe the ecology and biogeography of modern phytoplankton enable us to evaluate the ecosystem consequences of these phytoplankton radiations. In particular, our models suggest that the radiation of mixotrophic dinoflagellates and the subsequent diversification of marine diatoms would have accelerated the transfer of primary production upward into larger size classes and higher trophic levels. Thus, phytoplankton evolution provides a mechanism capable of facilitating the observed evolutionary shift in Mesozoic marine animals.

## 1. Introduction

In 1977, Geerat Vermeij [1] documented a pattern of widespread and persistent evolutionary change among benthic invertebrates in Mesozoic (252–66 Ma) oceans, a transformation he christened the Mesozoic marine revolution and interpreted in terms of increasing selection pressure by durophagous predators. This explanation suggests classic top-down control of ecosystem composition, but Vermeij recognized that the most straightforward way to increase predator abundance would be to boost primary production, adding a critical bottom-up component to his argument (later expanded and formalized as the theory of escalation [2,3]). Estimating the productivity of ancient oceans is challenging [4,5], with some of the most compelling, if indirect, arguments for secular increase in primary production coming from patterns of marine animal diversity, the very thing one would like to explain [6,7]. Here, we take a complementary approach and ask how animals in marine ecosystems would be affected by a change in the *composition* of primary producer communities. Our thesis is that observed Mesozoic changes in the composition of continental shelf phytoplankton can indeed help us to understand Mesozoic marine animal evolution.

## 2. Patterns of animal evolution in Mesozoic oceans

Vermeij [1] insightfully applied ecological variations detected in space to illuminate evolutionary patterns observed through time. Specifically, he noted that the claws of predatory crabs in the tropical Indo-Pacific region have greater crushing strength than those in the Western Atlantic Ocean; concomitantly, Indo-Pacific gastropods have thicker shells, more prominent defensive ornamentation, and coiling patterns better able to withstand compressive forces [8]. Indeed, Vermeij [9] argued more generally that spatial variations in the abundance and armament of shell-crushing predators covary with patterns of skeletal morphology in prey organisms.

Vermeij's arguments about Mesozoic marine evolution focused, in the first instance, on the gastropods so central to his ecological observations. Planisprial and open-coiled shells are common in upper Paleozoic and Triassic rocks; most have wide apertures and minimal ornamentation. Beginning in the Jurassic, however, such forms were increasingly supplanted by taxa with coiling more resistant to crushing, narrower and sometimes toothed apertures, and prominent spines and other ornaments—all recognized as morphological ways to avoid or survive durophagous predation. Underpinning the evolution of spines was the physiological ability to resorb and remodel shell carbonate during growth, a capacity widespread in younger but not older gastropods [1]. Continuing ecological research has strengthened the view that in modern oceans gastropod shell form varies as a function of predator pressure (e.g. [10–13]).

Other molluscs show comparable evidence for increased predation in Mesozoic oceans. Ammonites, for example, record an increasing incidence of shell repair in younger Mesozoic rocks [14,15], and ecological research confirms that shell repair structures faithfully record predator pressure [16]. Bivalves commonly escape predators by living infaunally. While bivalves evolved the ability to burrow early in their evolutionary history [17], most Paleozoic taxa were epifaunal or semi-infaunal [18]. Triassic and Lower Jurassic rocks are full of epifaunal bivalves, especially oysters and their relatives, but later in the Jurassic and continuing into the Cenozoic, bivalve assemblages increasingly became dominated by infauna, with epifaunal bivalves either motile (which facilitates predator avoidance [19]) or, save for the massively calcified rudists, limited to habitats where salinity or physical parameters inhibit predator populations [18,20–22].

Echinoderms also show both morphological and ecological responses to increasing predation. Stalked crinoids, ecologically important components of Paleozoic shelf and platform faunas, increasingly became limited to deeper habitats where predation is less common [23]; at the same time, crinoids in shallow marine environments increasingly evolved motility [24,25]. Brittle stars also became less abundant in shallow water environments, at least partly because of increased predator pressure [26,27]. Through the Mesozoic Era, skeletons of epifaunal echinoids exhibited both increasing mechanical strength and more conspicuous defences, especially spines, while infaunal echinoids radiated across shelves [28]. Once again, there is evidence for increased predation on crinoids within Triassic oceans [24], but this does not obviate the sweep of morphological and behavioural shifts observed from the Jurassic onward. Brachiopods also evolved increasing ornamentation in earlier Mesozoic oceans, but because options for defence enhancement were limited, most clades eventually declined in abundance and diversity [29,30]. Even calcifying red algae changed morphologically in the face of increased grazing by durophagous herbivores [31].

It is worth noting criticism of the Vermeij hypothesis, particularly a statistical analysis of diversity dynamics by Madin *et al*. [32], whose analysis recovered secular changes in diversity among infauna, motile epifauna, sessile epifauna, and carnivores consistent with those expected by Vermeij, but who argued for the statistical independence of these patterns. Rebuttals ([33–35], but see also [36]) have challenged the taxonomic, temporal, and spatial scales of this criticism, a key point being that broad scale diversity trends shed limited light on hypotheses about specific morphological features and behaviour.

In general, then, a persistent pattern of evolution characterizes skeletal organisms across several phyla in Mesozoic continental platform and shelf environments, and as Vermeij ([1], see also [37]) proposed, this pattern is parsimoniously explained by an increase in the abundance, size, and/or armament of the animals that preyed on these organisms. Fossils provide direct support for a Mesozoic–Cenozoic radiation of durophagous predators. Durophagy evolved long before the Mesozoic marine revolution (e.g. [38]) but shell-crushing fish [1], tetrapods [39], crustaceans [40], and predatory gastropods [41], asteroids [42], and echinoids [25] all show evidence of later Mesozoic and Cenozoic diversification. More generally, the proportional diversity of predators in among marine fossils has increased through the past 150 Myr [43,44], as have both the incidence of drill holes and repair scars on fossil skeletons [45] and crushed shell debris [46].

## 3. A second Mesozoic marine revolution

**Figure 1.**
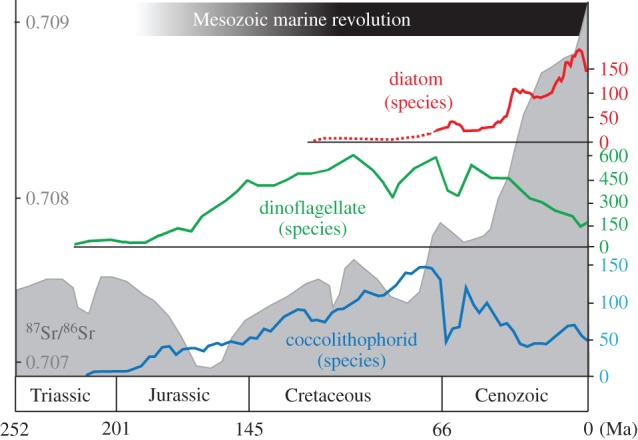
The Mesozoic marine revolution occurred during an extended interval of significant evolutionary change in marine primary producers, including radiations of photosynthetic dinoflagellates (green), coccolithophorids (blue) and, subsequently, diatoms (red). Strontium (Sr) isotopes (grey) suggest a significant enhancement of weathering and nutrient enrichment of the global ocean on the same time scale as (potentially related) diatom diversification. Microfossil diversity replotted from Falkowski *et al.* [48], based on original tabulations from Spencer-Cervato [49], Bown *et al.* [50], and Stover *et al.* [51]; strontium isotope data from Veizer *et al.* [81].

Today, diatoms, dinoflagellates, and coccolithophorids dominate primary production in continental shelf waters, and these are also the most abundant and diverse eukaryotic phytoplankton in the blue-water oceans [47]. All rose to ecological prominence in Mesozoic oceans and none is reliably recorded from earlier seas, where cyanobacteria and green phytoflagellates appear to have predominated ([48]; figure 1). This phytoplankton makeover is recorded by the biomineralized skeletons of diatoms and coccolithophoroids and by organic-walled dinoflagellate cysts. These records are potentially subject to preservational bias through time—one might imagine, for example, that early diatoms were only weakly mineralized, or that early dinoflagellates did not form recognizable cysts, obscuring an evolutionary history far longer than that recorded by microfossils. Steranes and other molecular biomarkers, however, provide a second record of phytoplankton evolution that largely corroborates the one reconstructed from microfossils [53–55], suggesting that marine sediments faithfully record a Mesozoic revolution in phytoplankton composition. Neither do molecular clocks suggest long prehistories for these clades [56–59]. Unambiguous dinoflagellate microfossils first appear in upper Triassic rocks and the group radiated through the Jurassic, reaching a diversity maximum in Cretaceous oceans, and much the same is true of coccolithophorids [60]. Diatoms diversified later, during the Late Cretaceous and, especially, Cenozoic [49,61–63].

Radiating Mesozoic clades differ from Paleozoic phytoplankton dominants in a number of traits. Dinoflagellates and diatoms commonly have larger cells than those of Paleozoic cyanobacteria and green algae. Diatoms and coccolithophorids are armoured, and, importantly, dinoflagellates are commonly mixotrophic. How might observed changes in the phytoplankton have influenced the faunal events identified by Vermeij?

## 4. A trait-based perspective on phytoplankton evolution

Current theoretical and modelling understanding of the functional and taxonomic biogeography of marine phytoplankton focuses on traits and trade-offs. Understanding and quantifying the key costs and benefits of a particular trait allows us to build diagnostic and predictive models. As an example, nitrogen (N) fixation relieves nitrogen stress in certain environments but has a high energetic cost, largely associated with oxygen management to protect nitrogenase; this cost reduces growth rates and growth efficiencies [64,65]. In addition, nitrogen fixers have a high iron demand to maintain the required nitrogenase [66]. With this understanding, using resource ratio theory, we can predict and interpret the biogeography of nitrogen fixation observed in today's oceans [67].

Key traits in any ecosystem include maximum growth rates, resource affinities, and defence characteristics. In a simplified model, consider the rate of change of biomass of phytoplankton phenotype *i* (*B_i_*, mol kg^−1^):4.1
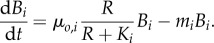
;We assume that Monod-kinetics appropriately describe resource-dependent growth, where *R* (mol l^−1^) is the limiting resource, *μ_o_*_,*i*_ (s^−1^) is the maximum growth rate, and *K_i_* (mol l^−1^) is the half-saturation. *m_i_* (s^−1^) represents all loss processes as a simple fixed rate. Two interesting limits reveal the significance of these traits for fitness. In a situation where resources are replete, net *per capita* rate of population increase depends on maximum growth and loss rates:4.2
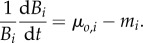
;Resource replete conditions are almost always intermittent and in such situations, over several cycles of replenishment, selection will favour the highest *per capita* growth rate that can be achieved by high maximum growth rate or good defence against losses. By contrast, in a steady state where nutrients are consistently depleted, the solution of (4.1) predicts that the subsistence resource concentration of phenotype *i* will be defined by its traits as follows:4.3
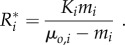
;The competitive exclusion principle suggests that, at equilibrium, the population with the lowest R* will exclude all others that are limited by the same resource [68,69]. Hence, maximum growth rate and mortality (defence) are key traits in both resource replete and oligotrophic conditions. Resource affinity (and *K_i_*) is also significant in the latter case.

How were these traits affected by the Mesozoic innovations among primary producers, and how would this have affected the structure of marine communities? Could these innovations have stimulated from the bottom-up some of the changes observed at higher trophic levels? To address these questions, we first discuss the allometric and functional structuring of key traits and then construct a simple numerical model of plankton population structure and productivity with which to explore several hypotheses.

### (a) Allometric constraints on productivity

**Figure 2.**
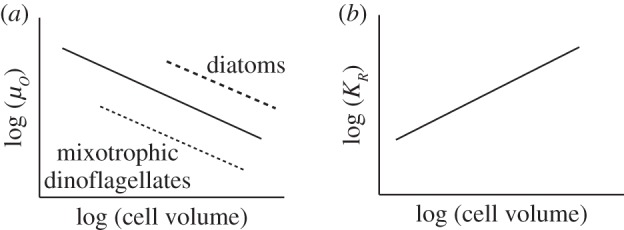
Schematic view of the power law relationships between cell volume and key traits of marine phytoplankton. These relationships are rooted in empirical observations and understood in terms of geometric effects on resource acquisition. (*a*) Maximum growth rate versus cell volume. The solid black line indicates the general trend used in the control model. Mixotrophic dinoflagellates (dotted line) follow the same trend but trade-off a lower growth rate against a generalist resource acquisition strategy. Diatoms (dashed line) are capable of faster maximum growth rates than other phytoplankton. (*b*) Resource half-saturation for the Monod-kinetics growth model versus cell volume.

Numerous studies have empirically demonstrated the scaling of reproductive rate with body size, showing a negative power law relationship from unicellular protists all the way to large mammals and trees. In eukaryotic phytoplankton, maximum growth rate (*μ_o_*_,*i*_, d^−1^) and cell volume *V*_i_ (μm^3^), follow the relationship 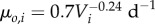 [70]: larger organisms have slower maximum growth rates. By contrast, nutrient half-saturation increases with cell volume: 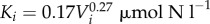 for nitrate-limited growth (figure 2).

Larger cells have both slower maximum growth rates and higher R*s. Thus, from a growth perspective, smaller cells should outcompete them everywhere. Indeed, the smallest primary producers are specialist gleaners that dominate the most oligotrophic marine environments. However, top-down control prevents them from sequestering all of an available resource, enabling populations of larger cells to coexist with grazer-controlled smaller cells [71–73]. If the maximum population size of primary producers is controlled by grazing (or viral losses), then, as the rate of resource supply increases, so too will the body size of the largest cells that can be supported.

This can be illustrated by extending the model above to include prey-specific predators and an explicit mass balance for the resource (described in box 1).

Box 1. **A simplified ecosystem model with allometric constraints on traits.**

**Figure 3.**
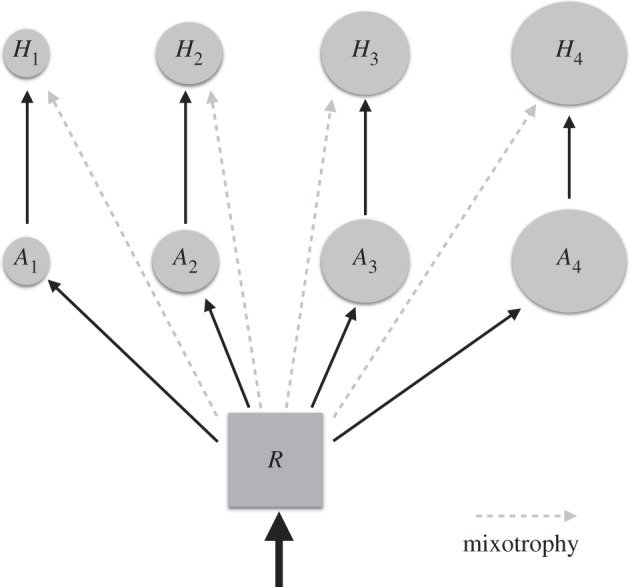
Schematic depiction of the simplified model employed here. A single inorganic resource, *R*, sustains an assemblage of photoautotrophs (*A_i_*) each of which is consumed by a specific predator (*H_i_*). Cell volume/body size increases with index *i*. Solid black lines indicate the flow of resource in the purely specialist (autotroph/heterotroph) model. Dashed grey lines indicate the additional flows when mixotrophy is introduced into the model.

**Figure 4.**
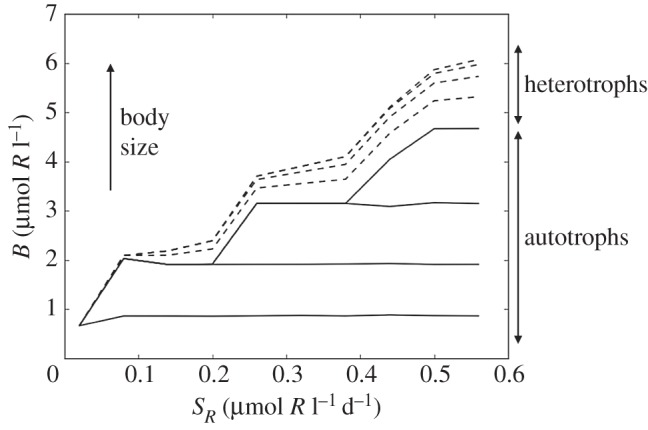
Cumulative biomass (*B*) with size as a function of resource supply rate (*S_R_*) in the ‘control’ model where maximum growth rate strictly follows the solid black line in figure 2*a*. The uppermost dashed line indicates total plankton biomass, summing the contributions from each size class of both autotrophs (solid lines) and heterotrophs (dashed lines) which are stacked with contributions from the smallest autotrophs at the bottom, and heterotrophs on top of autotrophs.

In equation (4.4), we define the governing equation for the rate of change of biomass of plankton type *i* (*B_i_*, mol m^−3^). Growth is represented by Monod-kinetics, limited by a single resource *R* (mol l^−1^), with maximum growth rate *μ_o,i_* (s^−1^), and resource half-saturation *K*_i_ (mol l^−1^). Cells can consume, and be consumed, by an arbitrary combination of the other plankton types (second and third terms on the right, respectively), both described as Holling II functional response models. Predatory gains are governed by a matrix of maximum growth rates, *g_oji_* ( (mol l^−1^ s)^−1^), where *ji* refers to type *j* consuming type *i*. Maximum grazing rates are set by the empirically informed, allometric power law: 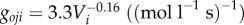 ([74], with assumptions as in [73]). Predatory losses are described similarly. *γ_ji_* is the efficiency with which consumed prey is converted to predator biomass. Finally, the fourth term on the right of (4.4) represents losses due to maintenance respiration and other, non-specific mortality. Equation (4.5) describes the community consumption of the inorganic resource, *R*, and its resupply, *S_R_*.4.4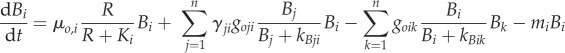;and4.5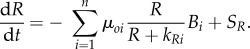;

Here we use a highly idealized model of the planktonic food web, depicted schematically in figure 3, to illustrate how size-dependent traits shape both the pattern of phytoplankton assemblages and the delivery of organic carbon to larger size classes. The model resolves four size classes of photoautotroph and four associated size classes of predatory heterotrophs. The mathematical framework is as described in box 1 and the traits (maximum growth and grazing rates, resource half saturations) are governed by the empirical power law relationships discussed above. The set of ordinary differential equations was integrated forward in time from an arbitrary initial condition to steady state for a range of rates of supply of the inorganic resource. As shown in figure 4, as resource supply increases so too does the capacity for larger primary producers and their predators to coexist with the smaller types. In figure 4, the lowermost solid line indicates the biomass of the smallest size class of primary producer as a function of nutrient supply. The next line indicates the cumulative biomass of the two smallest size classes and so on. Dashed lines indicate the contribution to total biomass from the associated predators in a similar way, and so the uppermost dashed line reflects the total standing biomass in the system as a function of the rate of delivery of inorganic resource. At the lowest resource supply rates, only the smallest phytoplankton classes are viable; they have the lowest R* and outcompete the larger cells but their own population remains too small to sustain a predator. However, as the nutrient supply increases so too does their population size until it reaches the subsistence level for their predators. This top-down control prevents further increase in the population size of the smallest autotrophs and caps their rate of resource consumption so that at even higher resource supply rates, some resource is available to larger size classes, which grow in until they also become subject to predation, and so on. This stacked relationship among size classes in the plankton is observed in the ocean today (e.g. [73,75]), providing empirical support for the mechanistic model. The simple framework can be adapted to represent more complex food webs with richer interaction networks, but they retain the same qualitative structure and implications [67,71].

**Figure 5.**
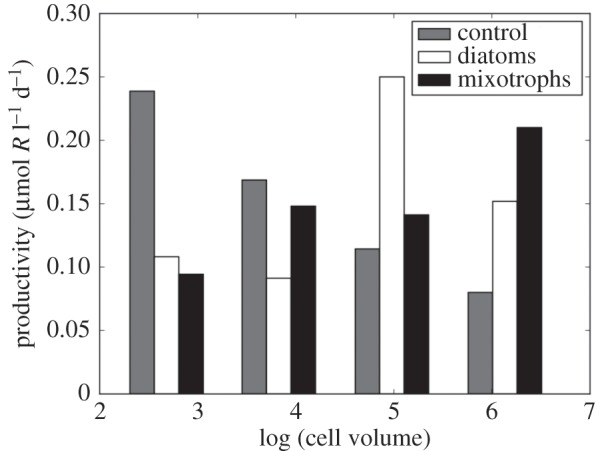
Total system productivity (primary and secondary) as a function of size class in the model at the highest resource supply rate shown in figure 4. Grey bars indicate the control solution where maximum growth rate strictly follows the solid black line in figure 2*a*. White and black bars indicate the model into which diatoms and mixotrophy were introduced, respectively, as described in the text.

The size distribution of biomass is rather flat (not illustrated), due to the top-down control by grazing, consistent with observed size spectra [76]. Here, however, we are concerned with the flow of material and energy up to the larger organisms that ultimately depend on these primary producers. The grey bars in figure 5 illustrate total productivity (autotrophic and heterotrophic; mol l^−1^ d^−1^) in each size class of the model at the highest nutrient supply rate shown in figure 4 where all four size classes coexist. Trophic transfer efficiency is low (here assumed to be 10%), and so in this ‘control’ model, where the traits exactly follow the allometric scalings, total productivity declines rapidly with increasing size. Since predatory plankton tend to consume organisms about an order of magnitude smaller than themselves [74], the biomass and productivity of the smallest cells is not directly accessible to large predators. The rapid decline in productivity with size means that the upwards flow of resources is relatively small, limiting productivity and population size higher up the food chain.

Hence, in a system where allometric constraints on resource affinities entirely dictate the assemblage, the delivery of material to larger size classes and higher trophic levels is very low. However, modification of the allometric relationships by alternative trophic strategies and physiological innovations can relieve this constraint. We illustrate two such mechanisms in the context of the model below.

### (b) Mixotrophy

Evolutionary innovations have modified the traits of primary producers, fuelling greater productivity in larger size classes which, we hypothesize, contributed a bottom-up stimulus for Mesozoic animal evolution. Not all phytoplankton lie on the same size-growth rate relationship (solid line in figure 2*a*). Notably, mixotrophic dinoflagellates trade-off the benefits of a generalist (autotrophic and phagotrophic) approach to nutrition against a slower maximum growth rate, size for size, relative to pure autotrophs (figure 2*a*, dashed line; see [77]). As large cells with inefficient resource acquisition and, hence, low growth rates, dinoflagellates would appear to compete poorly against other phytoplankers; however, because they can gain nutrients by phagocytosing other cells, mixotrophic dinoflagellates are both abundant and diverse in contemporary oceans. That is, mixotrophy allows larger cells to supplement resources for which they are less competitive (i.e. have higher R*) with respect to the inorganic form. This enables primary production of new organic material in larger size classes and enhances the flow of organic resources to higher trophic levels [78]. In a simple demonstration of this effect, we introduced mixotrophy into our model by allowing the heterotrophs to also grow autotrophically (i.e. assume a mixotrophic lifestyle) but with much reduced uptake rates (approx. 55%) for inorganic resources relative to the specialists. In figure 5, the black bars illustrate the resulting size structuring of productivity: the introduction of mixotrophy leads to a significant enhancement of productivity in the largest size classes, relative to the control case. In this highly simplified model, total productivity is greater in the larger size classes when mixotrophy is active. The general principle is borne out in more complex global ocean ecosystem simulations [74]. Hence, the radiation of mixotrophic dinoflagellates may have significantly altered the structure of marine productivity, providing bottom-up fuel for the Mesozoic marine revolution (figure 1).

### (c) Diatoms

Later on (figure 1), the diversification of marine diatoms opened up a new niche for highly effective opportunists. Size for size, diatoms have higher maximum growth rates than other phytoplankton, possibly related to the cost-effectiveness of building a silica-based frustule [79]. The frustules also provide effective defence. Hence, diatoms innovated both an enhancement to *μ_o_* and a reduction in *m*, improving their relative fitness both in boom-bust and stable, oligotrophic environments. In a further sensitivity study with the model, we examined the impact of enhancing the maximum growth rate of the two largest size classes (dashed line, figure 2*a*), mimicking the evolution of large, fast-growing diatoms (now in the absence of mixotrophy). The impact on the size dependence of productivity is shown in figure 5 (white bars). Higher growth rates provide an advantage for the diatoms, increasing total productivity in the larger sizes and the rate at which resources can be delivered to even larger (but unresolved) size classes and higher trophic levels.

## 5. Discussion

Our simple model illustrates three key concepts relevant to the Mesozoic marine revolution. Firstly, the model shows that the Mesozoic radiation of mixotrophic dinoflagellates would have enhanced the flow of resources to larger size classes and higher trophic levels (figure 5). Secondly, any increase in the rate of nutrient supply to the surface ocean would have opened a niche for larger primary producers (figure 4); packaging phytoplankton into larger cells will shorten food chains at the lower end, again enhancing upwards resource flow. And thirdly, the opening of that niche may have facilitated the rise of large, silicified diatoms, whose adaptation for fast growth rates in replete environments would have further accelerated the enrichment of higher trophic levels (figure 5). Thus, these interconnected events could underpin the evolutionary trajectories observed among marine metazoan fossils.

A predicted result of dinoflagellate radiation is the shortening of food chains, delivering more energy and biomass to the predatory populations at the apices of food webs [78]. Even in small amounts, mixotrophy should enhance community productivity [52]. We suggest that this enhancement of resource delivery to larger size classes and higher trophic levels contributed a bottom-up push to the Mesozoic marine revolution, providing fuel for the ensuing arms race between the consumers of primary producers and their predators.

Our current understanding of the size structuring of plankton biomass makes a clear case that larger size classes, with low nutrient affinities, are largely excluded from regions of low nutrient supply. Conversely, enhanced nutrient supply fuels a growing in of larger organisms (e.g. figure 4). Could a global-scale enrichment of ocean nutrients have driven a parallel restructuring of phytoplankton on a similar scale? As noted above, quantitative estimates of ancient primary production are hard to come by, and existing geochemical proxies commonly target export production, which has a complicated relationship to primary production in surface waters [4,80]. To the extent that nutrient fluxes from continental weathering and erosion regulate primary production in the oceans, one might assume that geochemical proxies for run-off should provide at least a qualitative indication of changing primary production through time. Thus, increasing ^87^Sr/^86^Sr, an indication of increasing continental input of Sr to seawater, relative to hydrothermal sources, should correlate with increasing phosphorous (P) fluxes into the ocean. A well-resolved record of seawater strontium isotopes has been constructed from analyses of skeletal carbonates [81]; this record suggests that primary production might well have increased nearly monotonically from the later Cretaceous Period to the Neogene, concomitant with the rise of diatoms to ecological prominence. Long-term secular trends are less obvious in earlier Mesozoic oceans; ^87^Sr/^86^Sr values do not exceed Triassic to earliest Jurassic maxima until the end of the Cretaceous Period (figure 1). Seawater strontium isotopes reflect the lithologies of eroding continental rocks as well as the amount of run-off, complicating attempts to quantify erosional fluxes [82]. Nonetheless, sediment accumulation rates [83] and thermochronology [84] both reinforce the view that erosional fluxes in the oceans increased through the Cenozoic Era. Lithium (Li) isotopes have more recently been applied to questions of continental weathering, and these also corroborate the hypothesis of increased weathering fluxes through the Cenozoic Era, reaching a high steady state over the past 10 Myr [85,86]. Limited Li isotopic data are also consistent with lower weathering fluxes before the latest Cretaceous Period (e.g. [87]). Proxies for continental weathering and erosion are, thus, consistent with the hypothesis of increasing resource availability at the base of marine trophic pyramids over the last 80 Myr or so, helping to explain the persistence if not the initiation of predator-driven evolutionary trends among marine animals.

It has also been hypothesized that innovations in terrestrial evolution might have resulted in higher nutrient fluxes from land to sea. Specifically, Bambach [43] hypothesized that flowering plants would have increased nutrient fluxes to the oceans, beginning in the mid-Cretaceous Period; however, Boyce & Lee [88] subsequently showed that the timing of angiosperm radiation fits poorly with patterns of Mesozoic marine evolution. On the other hand, seagrass and mangrove communities would have provided nutrient-rich nurseries for coastal animals from the Late Cretaceous onwards [89,90].

Diatoms were not part of the earliest Mesozoic phytoplankton radiations, but beginning in the Cretaceous Period and accelerating into the Early Cenozoic Era, they diversified to become major primary producers in productive ocean waters. In light of the hypothesized increase in continental run-off and, thus, nutrient enrichment, the radiation of phytoplanktonic diatoms can be interpreted in terms of the models described above. Increasing primary production would have facilitated the evolution of larger phytoplankton cells, opening a niche for diatoms, perhaps especially at high latitude sites of strong upwelling [91]. Cermeño *et al*. [62] have, in fact, proposed that the dissolved silica levels needed to support high diatom production are themselves a product of increased continental weathering and erosion. Large diatom cells, in turn, would have shortened food chains, increasing the flux of energy to top predators. That is, by shortening food chains, diatoms may have amplified the ecosystem consequences of increasing primary production. Moreover, limited ecological experiments suggest that bivalves fed on diatom-rich diets grow faster than those fed on green algae [92,93], supporting the hypothesis that the carbon (C) : N : P of diatoms (and coccolithophorids) promotes more efficient growth of grazers, again moving more energy upward through food webs [94,95].

In short, evolutionary changes in the composition of phytoplankton could have enabled much of the observed Mesozoic marine revolution among animals, whether or not net primary production changed through time. We note that coccolithophorids, the third component of the Mesozoic phytoplankton radiation, have not figured strongly in our perspective because their cells are neither large nor strongly mixotrophic. Coccolithophorids could, however, have contributed to Mesozoic ecosystem change to the extent that their mineralized scales served to facilitate export production, increasing remineralization depth and, through this, phosphate availability and, in consequence, primary production [96]. The radiations of both diatoms and coccolithophorids had signal biogeochemical consequences, not only increasing rates of organic matter export from surface water masses [96], but also changing the marine carbonate [97] and silica [98] cycles.

If radiating phytoplankton fuelled faunal change in Mesozoic oceans, what facilitated Mesozoic phytoplankton evolution? At present, this question has no definitive answer, but various lines of evidence hint at the right direction. Molecular clock estimates suggest that photosynthetic stramenopiles [99] and haptophytes [100] originated during the Neoproterozoic Era, long before the specific radiations of diatoms and coccolithophorids. Similarly, dinoflagellates appear to have Neoproterozoic origins, although whether early members of the clade were photosynthetic is less clear [101]. Such considerations suggest that Mesozoic phytoplankton radiations reflect specific innovations within already extant clades, environmental changes that favoured these clades, or both.

In one view end-Permian mass extinction facilitated the rise to ecological prominence of chlorophyll *a*+*c*−-bearing phytoplankton [102,103], either through selective survival or via the establishment of favourable environmental conditions during Triassic recovery. As Medlin [103] observed, however, whatever the role of end-Permian extinction, the subsequent ecological expansion of dinoflagellates and coccolithophorids must be understood in terms of physiological characters that promoted competitive success in Mesozoic oceans. Biomarker lipids document the continuing ecological importance of green algae through the Triassic Period [104].

Kooistra *et al*. [105] reviewed characters that underpin the ecological and evolutionary success of the diatoms, calling attention to pigments that capture a relatively broad and energetic portion of the visible light spectrum, highly efficient nutrient uptake, a vacuole capable of storing nitrate, and both physical (the siliceous frustule) and chemical defences against grazers. All may have played a role in the rise of the diatoms, but changing ocean chemistry and nutrient availability probably did as well. The case for increasing nutrient availability, beginning in the later Cretaceous Period and enhanced by long-term changes in ocean circulation and climate [60], has already been made, as has the corollary argument that increasing macronutrients would be accompanied by enhanced silica availability. Limited experiments support the view that diatom success reflects the interaction of biological innovation with environmental circumstance. For example, when Ratti *et al*. [106] ran competition experiments using selected diatoms, green algae, and cyanobacteria, the diatoms emerged as dominant in the present-day seawater, but were outcompeted by green algae in solutions designed to simulate seawater in mid-Paleozoic oceans.

Similarly resolved character analyses are not available for coccolithophorids and dinoflagellates, but they share a basic set of photosynthetic pigments with diatoms, and coccolithophorids, at least, share the presence of a biomineralized surface. Indeed, unlike animals, in which most innovations in skeletal biomineralization occurred in association with Cambrian diversification, planktonic protists show a Mesozoic peak in the first appearances of both siliceous and calcareous tests and scales [107]. This suggests increased Mesozoic predation pressure in parts of the food chain unassayed by Vermeij. It has also been observed that dinoflagellates, coccolithophorids, and diatoms have a lower iron (Fe) quotient than green algae and cyanobacteria, providing an advantage in increasingly well-oxygenated ocean basins [108], as well as enhanced growth at sulfate levels probably first sustained in Mesozoic oceans [106]. This issue deserves further study, especially as it is amenable to both experimentation (e.g. [106,109]) and exploration with suitable models [73,77,78,91].

## 6. Conclusion

Models originally articulated to explore phytoplankton ecology and biogeography in the present-day ocean provide a new perspective on ecosystem change in ancient oceans, supporting Vermeij's [1] proposal that top-down controls on Mesozoic marine evolution reflect bottom-up facilitation. The novelty of the viewpoint presented here lies in the argument that changes in the *composition* of Mesozoic primary producer communities, and not simply the *amount* of primary production, fuelled observed faunal changes documented by Mesozoic and early Cenozoic fossils. A combination of mechanisms may have enhanced and accelerated the flux of resources through primary producers to the trophic levels where the arms race chronicled by Vermeij took place. At the outset, a marked radiation of mixotrophic dinoflagellates may have accelerated the transfer of primary production upward into larger size classes and higher trophic levels. Then, nutrient enhancement by increased global rates of continental run-off likely boosted ocean productivity, enhancing productivity in larger size classes, and opening up ecological opportunities for diatom radiation. The high maximum growth rates of phytoplanktonic diatoms further accelerated the productivity of larger size classes, again promoting the flow of fixed carbon to higher trophic levels.

Thus, a combination of biogeochemical and evolutionary events conspired to make more resources available to middle trophic levels of the marine ecosystem, providing impetus for the Mesozoic marine revolution and its associated arms race. This perspective underscores the utility of considering palaeontological patterns of animal evolution within a broader ecological framework and indicates that ecosystem modelling can improve our understanding of the marine biota in time as well as in space.
